# Irrigation of peritoneal cavity with cold atmospheric plasma treated solution effectively reduces microbial load in rat acute peritonitis model

**DOI:** 10.1038/s41598-022-07598-2

**Published:** 2022-03-07

**Authors:** Mustafa Onur Oztan, Utku Kürşat Ercan, Ayşegül Aksoy Gokmen, Fatma Simsek, Gizem Dilara Ozdemir, Gökhan Koyluoglu

**Affiliations:** 1 Department of Pediatric Surgery, School of Medicine, Izmir Katip Çelebi University, Izmir, 35620 Turkey; 2 Department of Biomedical Engineering, School of Engineering and Architecture, Izmir Katip Çelebi University, Izmir, 35620 Turkey; 3 Department of Microbiology, School of Medicine, Izmir Katip Çelebi University, Izmir, 35620 Turkey; 4 Department of Histology, School of Medicine, Izmir Katip Çelebi University, Izmir, 35620 Turkey

**Keywords:** Biomedical engineering, Experimental models of disease, Paediatric research, Bacterial infection

## Abstract

Accurate and timely diagnosis of appendicitis in children can be challenging, which leads to delayed admittance or misdiagnosis that may cause perforation. Surgical management involves the elimination of the focus (appendectomy) and the reduction of the contamination with peritoneal irrigation to prevent sepsis. However, the validity of conventional irrigation methods is being debated, and novel methods are needed. In the present study, the use of cold plasma treated saline solution as an intraperitoneal irrigation solution for the management of acute peritonitis was investigated. Chemical and in vitro microbiological assessments of the plasma-treated solution were performed to determine the appropriate plasma treatment time to be used in in-vivo experiments. To induce acute peritonitis in rats, the cecal ligation and perforation (CLP) model was used. Sixty rats were divided into six groups, namely, sham operation, plasma irrigation, CLP, dry cleaning after CLP, saline irrigation after CLP, and plasma-treated saline irrigation after CLP group. The total antioxidant and oxidant status, oxidative stress index, microbiological, and pathological evaluations were performed. Findings indicated that plasma-treated saline contains reactive species, and irrigation with plasma-treated saline can effectively inactivate intraperitoneal contamination and prevent sepsis with no short-term local and/or systemic toxicity.

## Introduction

Appendicitis is the most common cause of acute abdomen that may require emergent surgical intervention during childhood^1^. The accurate and timely diagnosis of appendicitis in children can be challenging since symptoms frequently overlap with other conditions and young children cannot adequately describe the pain^2,3^. Delayed admittance or misdiagnosis of appendicitis may result in perforation, which has been reported in the literature at rates ranging from 27 to 51% depending on the age of the study population, even 100% in children under the age of three^4,5^. However, to prevent sepsis caused by peritonitis, immediate surgical intervention with adequate antibiotic therapy is required. Surgical management entails eliminating the focus (appendectomy) and reducing contamination. The reduction of peritoneal contamination has received much attention for a long time. Several authors have long recommended mechanical cleansing with swabs, but intraoperative irrigation has always been a source of contention^6^. The compound of the solution and the amount of the solution to be used and the various substances to be added to the solution, and even whether irrigation should be done are still unclear. Research is still being conducted to find the best type of irrigation solution.

Physical plasma is the fourth state of matter, which is produced as a result of the phase change of gas via ionization. Thus, physical plasma is also expressed as ionized gas and was first defined by Irving Langmuir as the new state of matter in 1927. As physical plasma is produced as a result of phase change, energy input to the gas for ionization is required. Such energy could be in the form of electrical energy. By supplying enough voltage to a gas between electrodes, plasma similar to natural plasmas such as sun, lightning, and aurora borealis can be obtained artificially^7,8^. When a high voltage is applied to a gas between electrodes, an electric field is created, which then causes the acceleration of free electrons in the gas. The accelerated electrons collide with gas atoms, and molecules and remove electrons from them, causing the gas to ionize. Plasma formation in an electric field could lead to the generation of thermal (hot) and non-thermal (cold) plasma based on the thermal equilibrium of the ionized gas and electrons. The temperature of thermal plasmas may reach up to thousands of Kelvins, and they can be used as an electrosurgical tool in the medical field. In non-thermal plasmas, while the electron temperature reaches thousands of Kelvins, the ionized gas remains at room temperature, allowing non-thermal plasmas to be applied to living organisms and tissues. The application of non-thermal plasma to living tissues has given rise to a novel scientific field called “Plasma Medicine”, which investigates the novel therapeutic effects of non-thermal plasmas^8,9^. Various uses of non-thermal plasmas have been reported in the literature, including decontamination, anticancer treatments, wound healing, dental applications, etc^10–13^. One of the most well-known biomedical applications of non-thermal plasma is its antimicrobial effect. During the formation of the plasma, various reactive oxygen species (ROS) and reactive nitrogen species (RNS) (collectively known as reactive oxygen and nitrogen species: RONS) with biocidal effects are produced^14^. Non-thermal plasma and plasma-treated liquids have been shown to have a potent antimicrobial effect on gram-negative and positive bacteria, including multi-drug resistant (MDR) strains, in their planktonic and biofilm forms^15^. Furthermore, plasma devices that are registered as medical devices are used to treat infected non-healing chronic wounds and show remarkable microbial inactivation on wounds^16^. Similar to the direct antimicrobial effect of non-thermal plasma, liquids that are treated by non-thermal plasmas can have a strong antimicrobial effect when it comes in contact with pathogens. When a liquid is treated with non-thermal plasma, it undergoes chemical modification and transforms into a potent antimicrobial solution. RONS that are generated during the treatment of a liquid could be accumulated in the treated liquid, and the liquid itself can generate antimicrobial species as a result of the reaction with plasma discharge^14,17^. Various liquids such as water, phosphate-buffered saline (PBS) solution, solutions of organic substance, and saline solution (0.9% NaCl solution) were found to exhibit a strong antimicrobial effect when treated with non-thermal plasma^17,18^. The antimicrobial effects of a particular plasma-treated liquid may show differences depending on the plasma source, as various researchers make use of different plasma sources. Furthermore, the antimicrobial strength of a plasma-treated liquid is affected by a variety of factors such as plasma treatment parameters, plasma treatment time, liquid volume, initial microbial load, contact time of the liquid with pathogens, and the liquid volume to the number of the microorganisms ratio^17,19^. Thus, an in-vitro evaluation of a plasma-treated liquid, including chemical and antimicrobial characterization, is important to use those liquids in vivo antimicrobial applications.

Although different plasma treatment methods including direct plasma treatment, indirect plasma treatment such as plasma jets and plasma treated liquids have been shown to be an effective antibacterial, antitumor, and anti-inflammatory agent in various animal experiments^12,20–22^. However, none of these plasma treatment methods has not been tested in cecal ligation and puncture (CLP)-induced intraabdominal sepsis. Therefore, the aim of this study is to evaluate the efficacy of plasma-treated saline (PTS) irrigation after CLP in an animal model. Saline solution is routinely used for the irrigation of abdominal cavity in case of the acute appendicitis. Thus, the saline solution was selected to be treated with plasma and test its effect on CLP-induced intraabdominal sepsis model^23^.

## Results

### Optical emission spectroscopy

**Figure 1:**
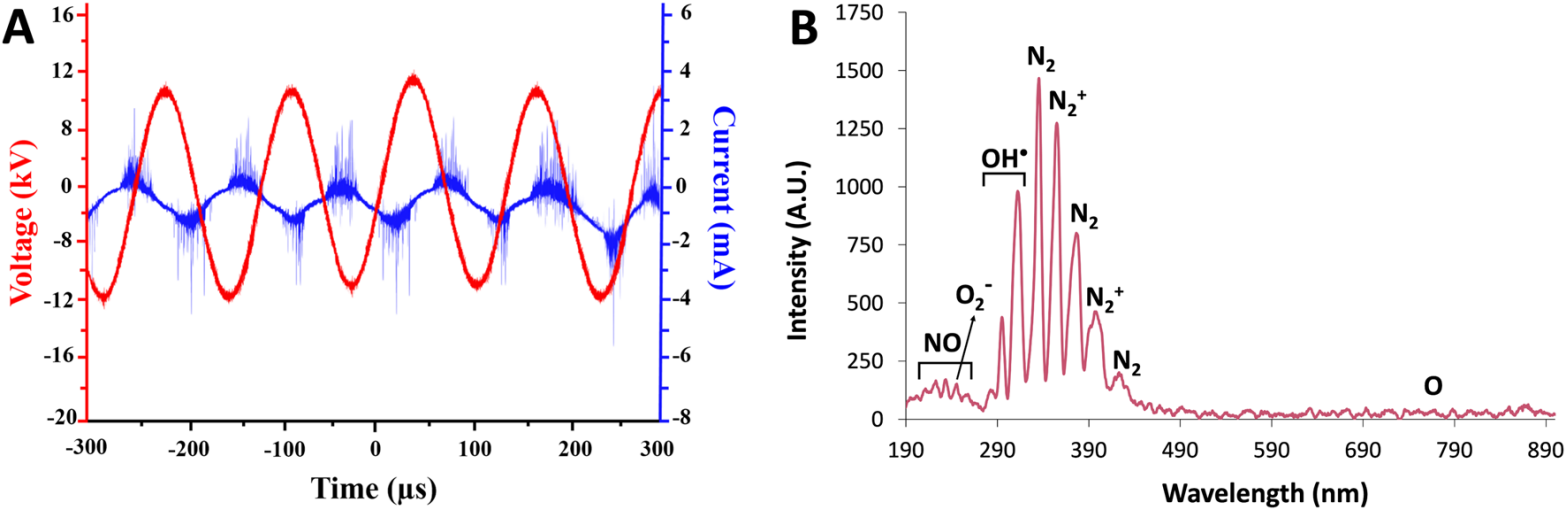
(**A**) Voltage-current waveforms recorded during plasma treatment of saline solution, (**B**) optical emission spectrum of the plasma discharge during treatment of saline solution.

Reactive species produced by plasma were detected using optical emission spectroscopy, and their spectra are shown in Fig. 1. High-intensity OH^·^ peaks around 309 nm and low-intensity peaks of singlet oxygen around 780 nm were observed. A peak at 254 nm that corresponds to superoxide anion was also observed. Furthermore, individual peaks at the 350–450 nm range that corresponds to emission from N_2_ and N_2_^+^ were seen. Furthermore, emission peaks at the 200–250 nm range can be linked to NO. Most of the emission was observed in the range of 300–400 nm, corresponding to the near UV region. Overall, the optical emission spectra of the pin-to-liquid plasma discharge used in this study showed the formation of ROS and RNS, both of which play important biological roles.

### Chemical characterization of PTS Solution

**Figure 2:**
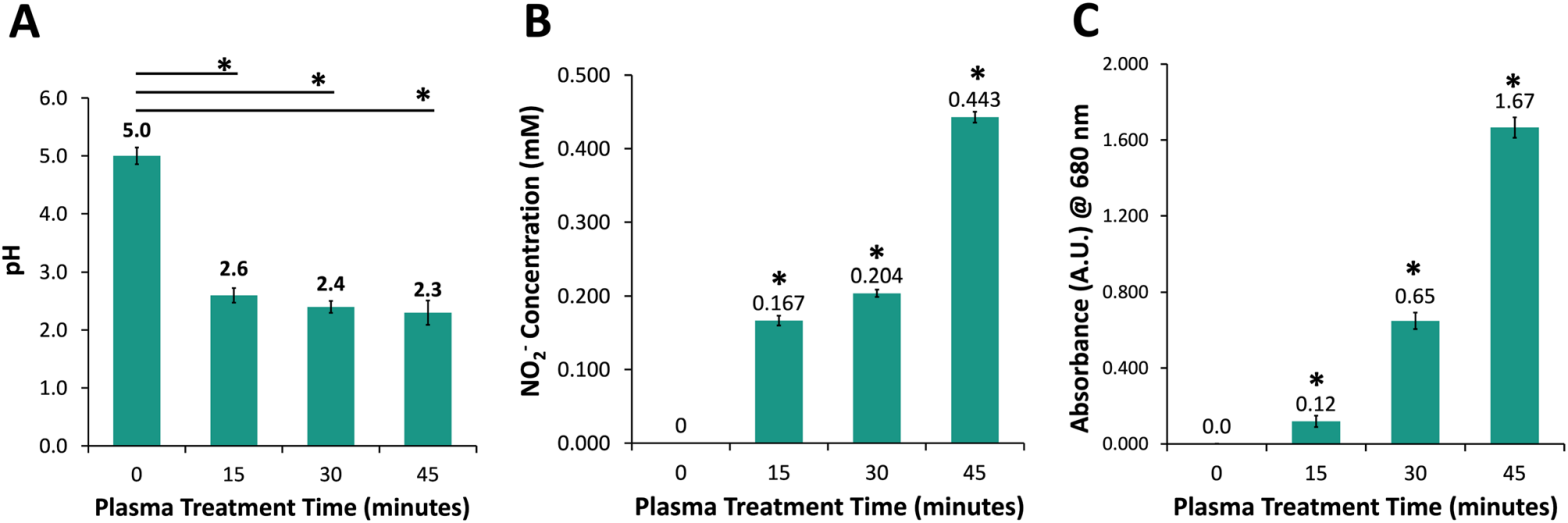
Chemical analysis of the plasma-treated saline solution (**A**) pH change of saline solution, (**B**) nitrite concentration in saline solution, and (**C**) total oxidative strength of saline solution determined by KI-starch assay with respect to plasma treatment time.

The chemical characteristics of the PTS solution revealed by the analysis including pH, nitrite concentration, and total oxidative species content measurements. As shown in Fig. 2A, the pH of the saline solution drops after plasma treatment. The pH of PTS was found to be 2.6, 2.4, and 2.3 for 15, 30, and 45 min treatments, respectively. The pH values of PTS for different treatment time points were significantly lower compared to untreated saline solution. Moreover, the difference in pH values of saline solutions treated for different timeframes was statistically insignificant (*p* > 0.05).

**Figure 3:**
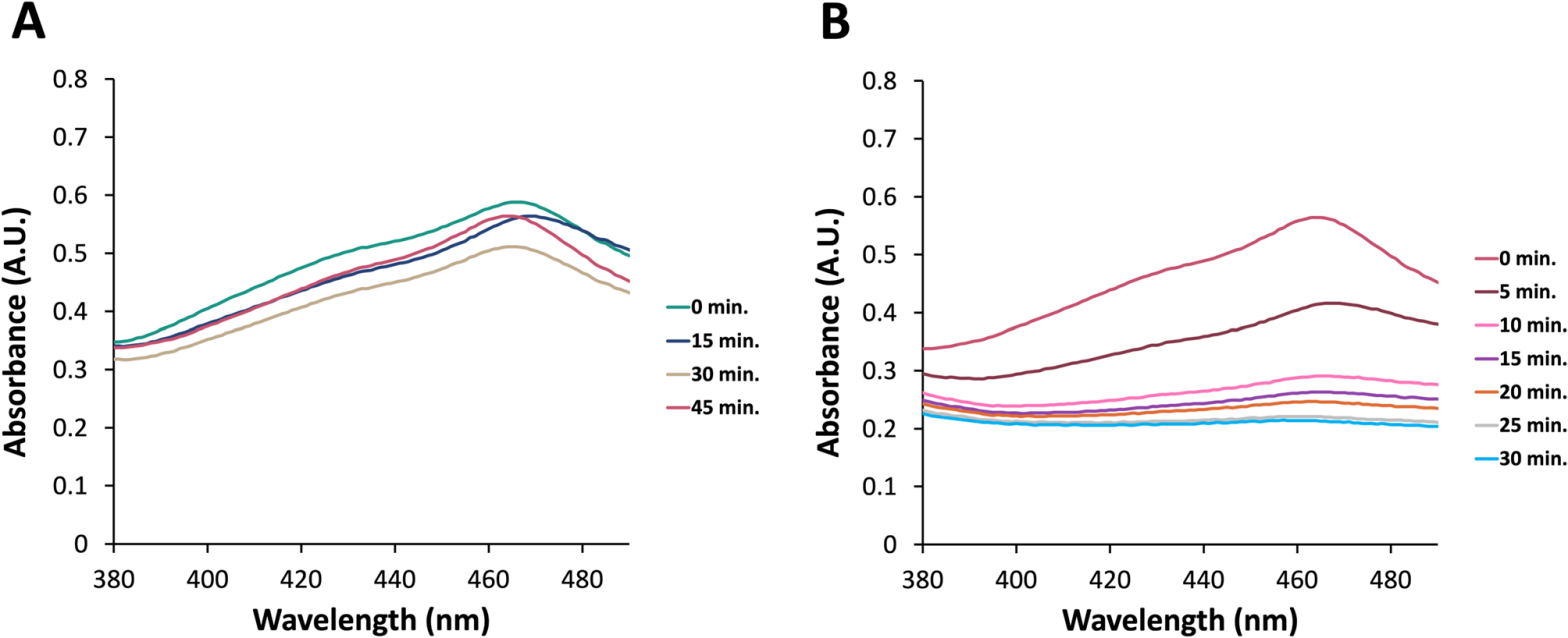
Detection of singlet oxygen in plasma-treated saline (**A**) With respect to plasma treatment time, and (**B**) After various time points following the completion of 45 min plasma treatment.

The nitrite (NO_2_‾) concentration in PTS increases with the duration of plasma treatment. This is evident from the study’s measurements of NO_2_‾ concentrations at 0.167, 0.204, and 0.443 mM for 15, 30, and 45-min plasma treatments, respectively, as shown in Fig. 2B. For all treatment time points, concentrations of NO_2_‾ were statistically significant. The total oxidative strength of the PTS was measured by mixing PTS with KI-starch reagent for which the absorbance value of 680 nm was found. The absorbance of PTS at 680 nm significantly increased with plasma treatment time and was measured as 0.12, 0.65, and 1.67 for 15, 30, and 45-min plasma treatments, respectively (Fig. 2C). Singlet oxygen in PTS was measured using DPBF through absorbance measurement and was found to increase with an increase in plasma treatment time similar to other detected species, as shown in Fig. 3A. Furthermore, the singlet oxygen content in 45-min plasma-treated PTS increases after undergoing treatment for up to 30 min, as the absorbance of DPBF decreases when singlet oxygen reacts with the reagent, as shown in Fig. 3B.

### In vitro antibacterial effect of PTS solution

**Figure 4:**
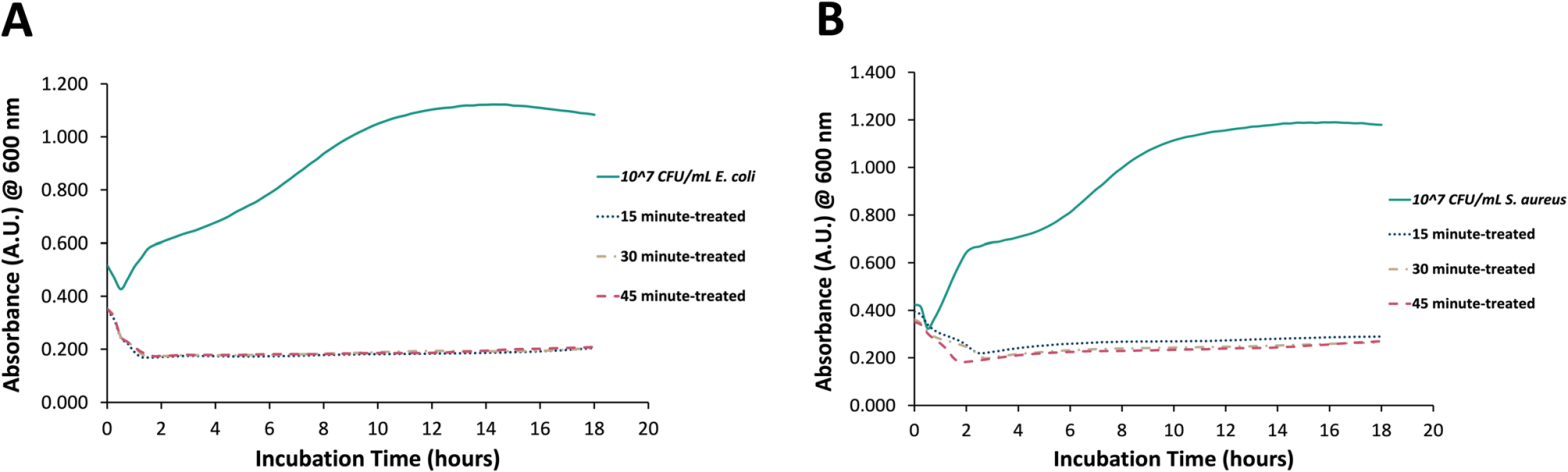
Kinetic bacterial growth curves. (**A**) *E. coli* and (**B**) *S. aureus* that were exposed saline solutions that were treated for 15, 30, and 45 min.

**Figure 5:**
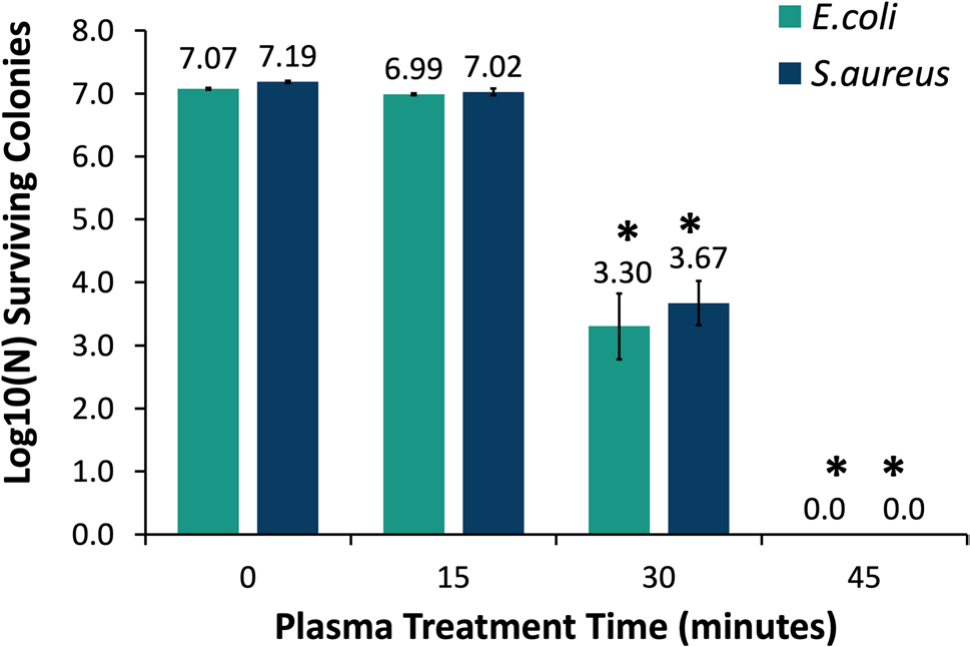
Antimicrobial effect of plasma-treated solution on *E. coli* and *S. aureus* with respect to plasma treatment time. The number of viable colonies were determined after exposure of pathogens to plasma-treated saline solutions for 2 h and determined with colony counting assay.

Growth kinetic measurement and colony count assay methods were used to evaluate the antibacterial effects of PTS on *Escherichia coli* (*E. coli*) and *Staphylococcus aureus* (*S. aureus*). As depicted in Fig. 4, control samples containing bacterial culture and untreated saline solution showed characteristic microbial growth, with both organisms in the stationary phase by the end of the measurement period. On the other hand, saline solutions treated with plasma for 15, 30, and 45 min led to the inhibited growth of *E. coli* and *S. aureus* in which the absorbance of samples at 600 nm did not show any increase over the 18-h measurement period. Later, a colony counting assay was carried out with PTS in order to determine whether the constant absorbance in growth kinetic measurement resulted from the inactivation by PTS or dormancy caused by the PTS-induced stress on bacteria. As displayed in Fig. 5, 15-min PTS did not lead to any significant bacterial inactivation on tested strains. On the other hand, 30-min and 45-min PTSs caused reductions of about 3.5 and 7-log, respectively, on *E. coli* and *S. aureus*, and these inactivation values were statistically significant (*p* < 0.05).

### In vivo microbiological evaluation

In microbiological culture analysis of blood, there was no growth in the Sham and Plasma groups. In the CLP group, there were 6 samples positive for *E. coli*, 2 for *Proteus vulgaris,* and 1 for *Klebsiella oxytoca* growth. In the CLP + Dry cleaning group, there were 6 samples positive for *E. coli* and 2 for *Proteus vulgaris* growth. In the CLP + saline group, there was 3 samples positive for *E. coli* growth. No growth was detected in the CLP + plasma group.

### Biochemical analysis

There were statistically significant differences in TAS, TOS, and OSI levels among the groups. When compared to Group Sham, mean TAS levels were significantly lower in Group CLP and CLP + Plasma (*p* < 0.05). In addition, mean TAS levels were significantly higher in Group CLP + Saline when compared to Group CLP (*p* < 0.05). There were statistically significant differences among the groups in TOS and OSI levels (*p* < 0.05). When compared to all other groups, mean TOS levels of Group CLP + Plasma were significantly lower. Moreover, when compared to Group CLP, mean OSI levels were significantly lower in Group CLP + Saline and CLP + Plasma. The mean values of oxidative and antioxidative parameters involving TAS, TOS, and OSI in the experimental groups, along with their SD, are shown in Table 1.

**Table 1:** Total antioxidant status (TAS), total oxidant status (TOS), and oxidative stress index (OSI) for animal experimental groups.

	Sham	Plasma	CLP	CLP + dry cleaning	CLP + S	CLP + plasma
TAS	1.55 ± 0.09	1.31 ± 0.19	1.23 ± 0.24^a,e^	1.27 ± 0.18^a^	1.52 ± 0.20^c^	1.25 ± 0.17a
TOS	23.19 ± 6.04	24.55 ± 7.59	33.22 ± 6.03	31.33 ± 8.58	24.77 ± 12.49	6.29 ± 3.19^a,b,c,d,e^
OSI	14.70 ± 3.55	19.01 ± 5.85	25.12 ± 8.39^a^	24.66 ± 5.33^a^	16.02 ± 7.00^c^	9.52 ± 8.78^c,d^

^a^Different from Group Sham.

^b^Different from Group Plasma.

^c^Different from Group CLP.

^d^Different from Group CLP + Dry cleaning.

^e^Different from Group CLP + S.

### Histopathological evaluation

**Figure 6:**
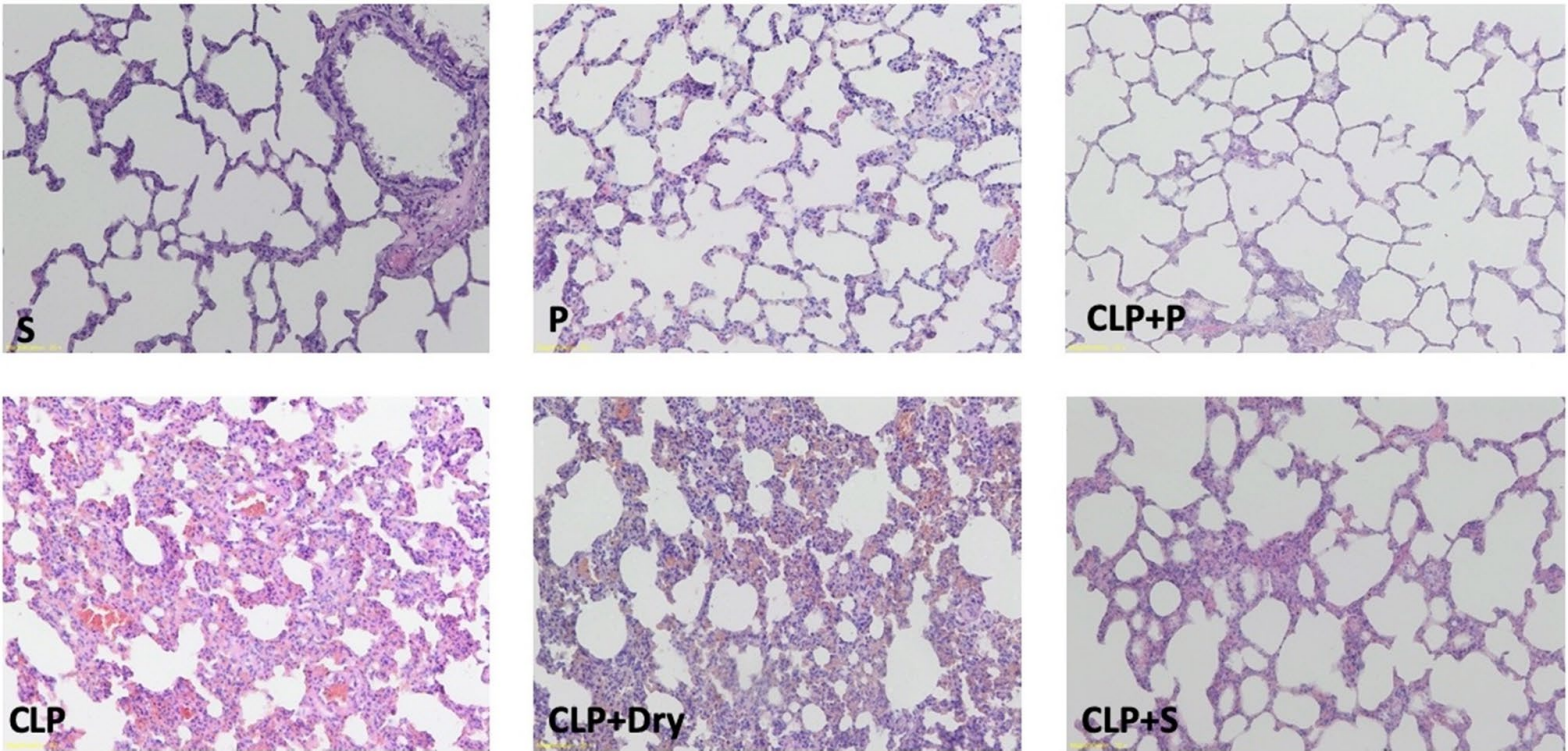
Representative histological examination images of lung tissue for each animal experimental group that were used for histological scoring.

**Figure 7:**
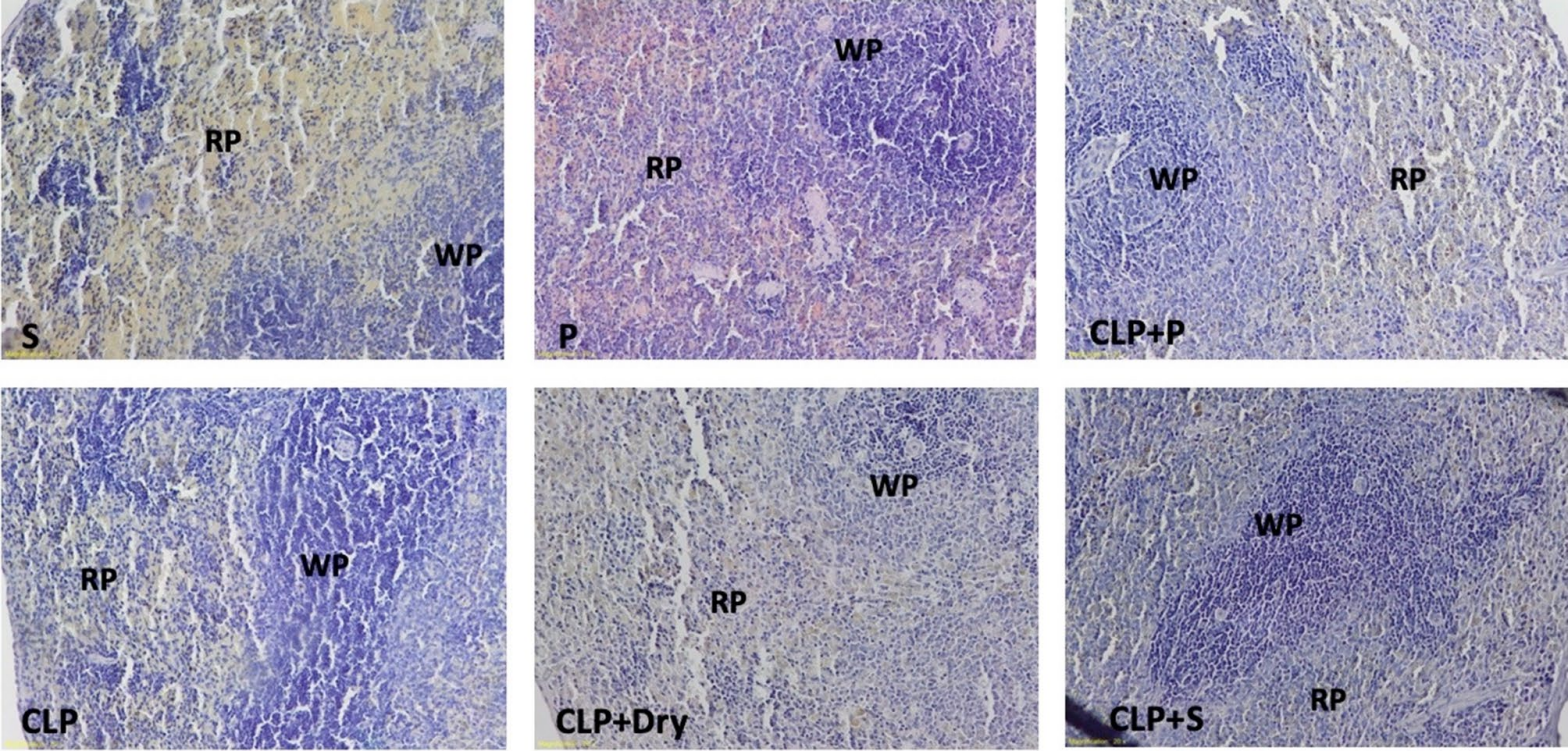
Representative histological examination images of spleen tissue for each animal experimental group that were used for histological scoring (WP: white pulp, RP: red pulp).

**Figure 8:**
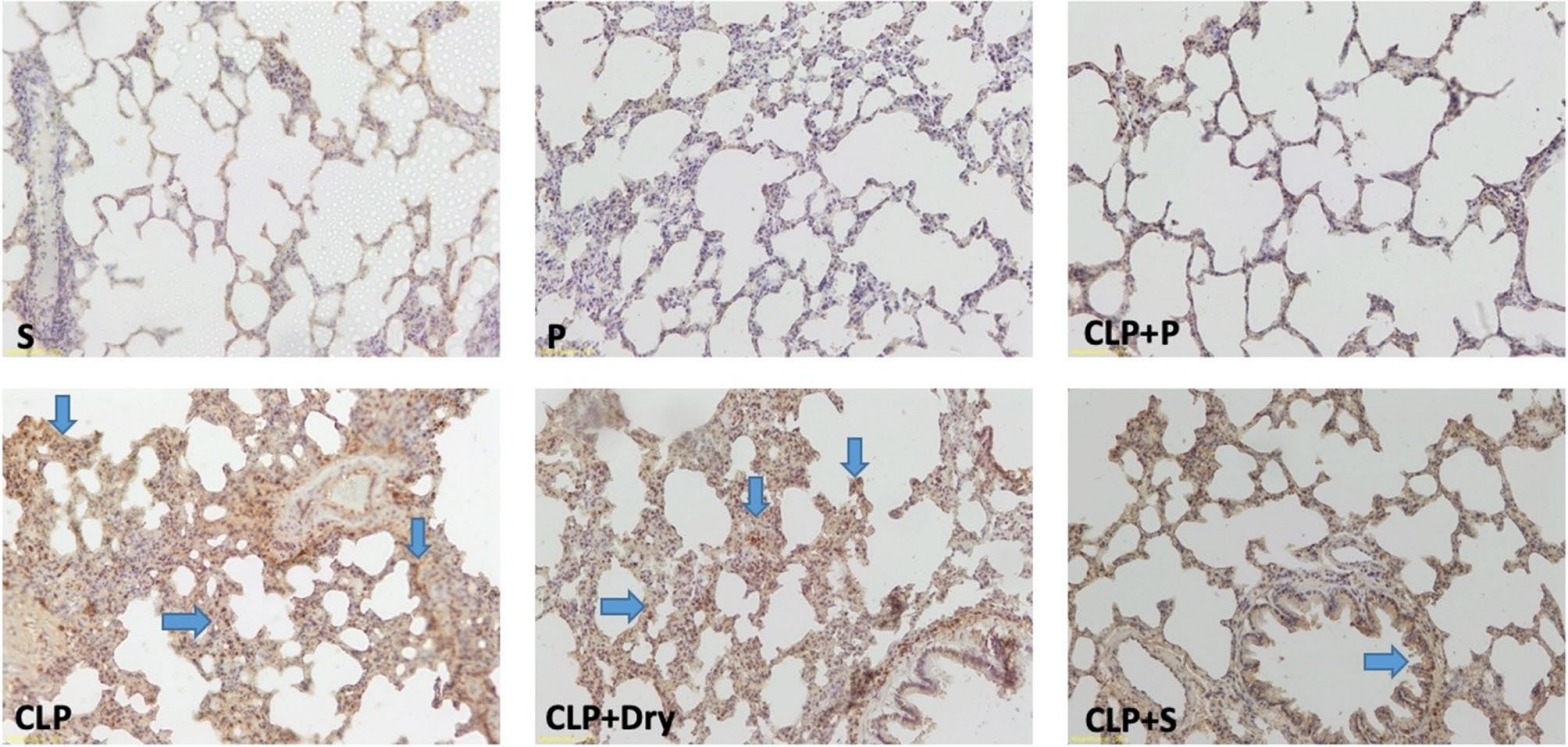
Representative histological examination images of lung tissue for each animal experimental group showing the extent of apoptosis that were used for TUNEL scoring.

**Figure 9:**
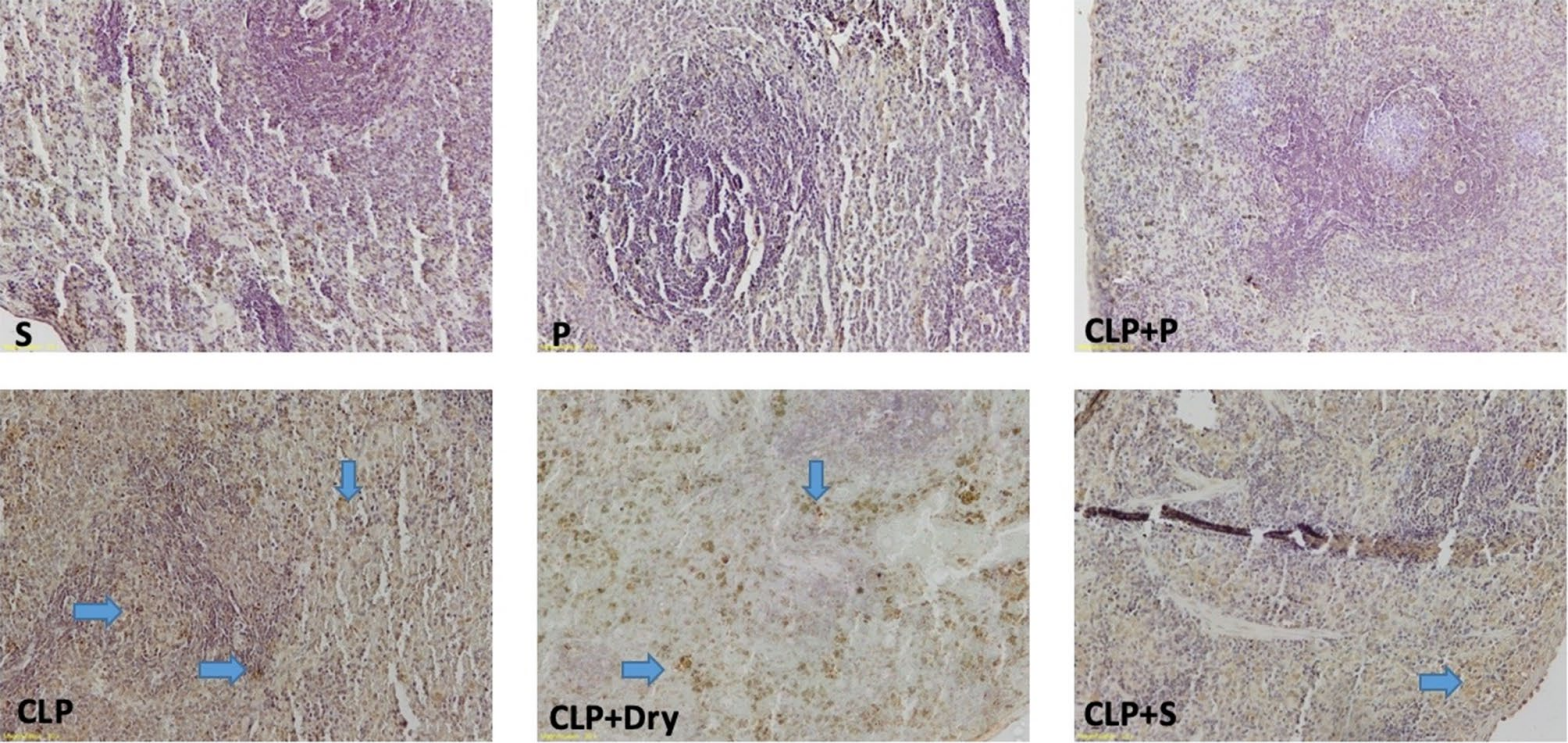
Representative histological examination images of spleen tissue for each animal experimental group showing the extent of apoptosis that were used for TUNEL scoring (WP: white pulp, RP: red pulp).

There were statistically significant differences among the groups in terms of injury in the lung tissue and inflammation scores of the spleen (*p* < 0.05). The values of Group CLP and CLP + Dry cleaning were higher than that of Group Sham. The values of Group CLP + Plasma were lower than those of Group CLP but were not different from those of Group Sham. Lung damage and spleen inflammation in Group CLP + Saline were more severe than those in Group Sham (Figs. 6 and 7). Regarding the TUNEL study, the positive cells in CLP and CLP + Dry cleaning groups were more intense than those in Group Sham. In Group CLP + Saline, the scores were higher than those in Group Sham but were also lower than those in the CLP group. In Group CLP + Plasma, scores were lower than the CLP, CLP + Dry cleaning, and CLP + Saline groups but no difference from Group Sham could be seen (Figs. 8 and 9). The mean values of lung and spleen histology and TUNEL scores in the experimental groups, along with their SD, are displayed in Table 2.

**Table 2:** Histology and TUNNEL scores of lung and spleen samples for animal experimental groups.

	Sham	Plasma	CLP	CLP + Dry cleaning	CLP + S	CLP + Plasma
Lung histology	0.56 ± 0.53	0.30 ± 0.48^c^	2.00 ± 1.05^a,b^	2.71 ± 0.49^a,b^	1.56 ± 1.0^b^	0.78 ± 0.83^c,d^
Spleen histology	0.29 ± 0.49	0.67 ± 0.52	2.50 ± 0.76^a,b^	1.83 ± 0.75^a^	2.11 ± 0.93^a,b^	0.60 ± 0.55^c,e^
Lung tunnel	18.25 ± 6.98	25.25 ± 5.39	61.25 ± 15.75^a,b^	70.71 ± 6.73^a,b,c,e^	38.75 ± 11.57^a,c,d^	26.25 ± 3.54^c,d,e^
Spleen tunnel	10.83 ± 3.76	20.83 ± 3.76	72.14 ± 8.59^a,b^	57.50 ± 9.35^a,b^	54.38 ± 15,91^a,b,c^	23.00 ± 5.70^c,d,e^

^a^Different from Group Sham.

^b^Different from Group Plasma.

^c^Different from Group CLP.

^d^Different from Group CLP + Dry cleaning.

^e^Different from Group CLP + S.

## Discussion

The purpose of this study was to explore the effects of PTS in rats with experimental peritonitis. To the best of our knowledge, the effects of PTS on experimental peritonitis have not been investigated in any model before. We initially designed our device to treat saline with plasma and optimized the treatment duration to kill enough bacteria. After obtaining adequate PTS, we demonstrated that lavage with PTS after cecal ligation and perforation reduced the severity of oxidative stress and the harmful effects of sepsis in distant organs.

A delayed diagnosis of appendicitis may result in complicated intra-abdominal sepsis in children. To remove necrotic tissues, intestinal contents, and bacteria from the peritoneal cavity, peritoneal lavage is used as a routine practice in many clinics for the treatment of secondary bacterial peritonitis. To reduce the morbidity and mortality related to peritonitis-related sepsis, the surgeon must control and clean the bacteremia source as much as possible. Despite numerous clinical and experimental trials with antibiotics, antiseptics, or different fluids, there is still no widely accepted formula.

Antibiotics were first used in peritoneal lavage in the late 1940s, but there is little clinical evidence that this treatment is effective^24^. In their meta-analysis work of 2010, Qadan et al. showed that antibiotic lavage demonstrated a significant reduction in mortality in experimental peritonitis^25^. Researchers are also widely investigating the use of substances other than antibiotics. Antiseptics such as chlorhexidine and povidone-iodine demonstrated promising results but side effects were also reported^26,27^. To avoid these side effects, the concept of using pure saline or enriched saline was developed. Kubota et al. with electrolyzed strong acid saline, Sada with hydrogen-rich saline, and Ozmen et al. with ozonated water showed promising results on CLP-induced sepsis in rats^28–30^. Based on these findings, we aimed to test the effects of PTS solution on the CLP-induced model of sepsis.

Plasma-treated liquids have antimicrobial effects on pathogens in both planktonic and biofilm forms, including multidrug-resistant (MDR) pathogens^15^. Although antimicrobial efficacies of plasma-treated liquids in vitro are well-known, evaluating these efficacies in vivo is crucial to indicate the effectiveness of those plasma-treated liquids for various potential applications in clinical practice. In the present study, the antimicrobial effect of PTS solution was evaluated in vitro and in vivo represents its potential use as an intraperitoneal irrigation solution. As the irrigation of the abdominal cavity requires higher volumes of liquid, the pin-to-liquid discharge plasma treatment (Fig. 1) was performed since it allows for the treatment of liquids of higher volumes^31^. The kinetic growth analysis of *E. coli* and *S. aureus*, which were used as model gram-negative and gram-positive strains in vitro antimicrobial experiments, respectively, revealed that PTS solutions treated for 15, 30, and 45 min could suppress the growth of tested strains. Previous studies reported that plasma treatment can induce viable but non-culturable (VBNC) states on bacteria, which can be caused by exposing the bacteria to a stressor where microorganisms can sustain their metabolic activities without cell division^32,33^. Therefore, to rule out the possibility of VBNC induction in *E. coli* and *S. aureus* by PTS solution, a colony counting assay was also carried out on those strains. The colony counting assay results showed an increasing bacterial inactivation with increasing plasma treatment time, which reached a complete inactivation (7-log inactivation) on exposure of a 45-min PTS solution to bacterial strains. The plasma treatment’s time-dependent increase in bacterial inactivation can be attributed to higher accumulation and/or generation of reactive plasma species such as ROS and RNS in the saline solution during this process^34,35^. This finding was consistent with the reactive species identified in the PTS solution. As the plasma treatment time increased, the pH of the PTS solution decreased. The pH value for each plasma treatment time point was statistically significantly lower compared to the pH of the untreated saline solution while there was no statistically significant difference between the pH of saline solutions that were treated for different time points. This finding suggests that the pH does not have a dominant role in the inactivation of the bacteria. The acidic pH is a well-known outcome of the plasma treatment of different liquids and was previously reported to be a critical feature for the antimicrobial effect of plasma-treated liquids, albeit not primarily responsible for this effect^14,36^. The optical emission spectrum recorded during saline solution treatment represents the formation of ROS and RNS in the gas phase, which play biological roles. The presence of nitrite and acidic pH indicates the formation of nitrous acid in saline solution as a result of plasma treatment. Furthermore, the peaks representing superoxide (O_2_^−^) and nitric oxide (NO) on OES could be interpreted as the formation of peroxynitrite (ONOO^−^), which was previously reported to be one of the dominant reactive plasma species responsible for the antimicrobial effect of the plasma-treated liquids^14,31,37^. The nitrite concentration, singlet oxygen content, and overall oxidative strength of the PTS solution increased with plasma treatment time, implying that ROS and RNS play a role in the antimicrobial effect of the PTS solution. The detection of singlet oxygen in the PTS solution was carried out using the DPBF reagent, which is a yellow-colored reagent that reacts with singlet oxygen to yield a colorless product^38,39^. Therefore, lower absorption of the DPBF, when exposed to plasma-treated liquid, reflects a higher amount of singlet oxygen. Despite the fact that the DPBF is commonly used to detect singlet oxygen, it can also react with other ROS such as superoxide (O_2_), hydroxyl radical (OH^·^), and hydrogen peroxide (H_2_O_2_)^40^. Although the absorption of the DPBF was found to be around 410 nm for the detection of ROS, in the present study, the peak of the DPBF was seen around 460 nm when DPBF was mixed with PTS solution. This shift of the peak could be a result of the acidic character of the PTS solution or the presence of various reactive species in PTS. Such speculations deserve a more detailed investigation. Furthermore, the amount of the singlet oxygen (or other ROS that react with the DPBF) increases with the time that passed after the completion of plasma treatment (Fig. 4B). Such findings can be attributed to the formation of hypochlorite (OCl^−^) in saline solution as a result of plasma treatment. The reaction of H_2_O_2_ with OCl^−^ results in the formation of the singlet oxygen^41^. Atomic oxygen, ozone (O_3_), hydroxyl radical (OH^·^), and hydrogen peroxide (H_2_O_2_) were previously reported as ROS that could oxidize the chloride (Cl^−^) molecule to form hypochlorous acid (HOCl) and hypochlorite (OCl^−^), both of which are potent antimicrobial chemical species^42^. In the in vitro studies, the saline solution was treated the day before the surgery on rats and stored at a temperature of 4 °C until surgery, which lasted for 18–24 h. Therefore, the antimicrobial effect of the 45 min PTS solution stored at 4 °C for up to one week was also evaluated. Before the antimicrobial tests on the stored samples, their temperature was brought to 37 °C since the plasma-treated samples used in in-vivo experiments were heated to 37 °C so as to prevent hypothermia in rats. The antimicrobial effect of the stored samples was comparable to that obtained with the freshly treated samples and was found to be 7-log.

The contamination of the abdominal cavity with intestinal contents is a polymicrobial contamination that almost entirely consists of *Enterobacteriaceae* and *Bacteroides* species^43^. The normal serosal mesothelium is a cell layer based on a basal membrane and elastic fibers. Edminston et al. showed that four hours post-CLP, the serosal mesothelium was colonized by the enteric bacteria, predominantly by *E.coli*, *Enterobacter*, and *Proteus* species^44^. Pross et al. showed that two hours post-contamination seven out of eight rats and eight hours post-contamination all of the eight rats had a positive blood culture of enteric pathogens, indicating bacteremia^45^. The dry cleaning technique has the advantage of the removal of the microbial content from the abdomen, but the interloop spaces, abdominal recesses, and pelvic depths may not be sufficiently cleaned most of the time^46^. According to the experimental and clinical studies, the use of simple saline irrigation is preferable to no lavage^23,47,48^. In adults, the important issue is to use high-volume intraoperative lavage, typically 8–12 L of physiological saline^49^. In a preliminary report, Ohno et al. reported that 6 L/m^2^ lavage fluid has an effect of decreasing bacteria in the abdomen of children^50^. In our CLP, CLP + Dry cleaning, and CLP + Saline groups, there are 9, 8, and 3 positive blood cultures of enteric microorganisms, respectively. Our results show that PTS significantly reduces the bacteremia rate due to a lack of growth in our CLP + Plasma group. Such a result could be attributed to PTS reaching all spaces in the intraabdominal cavity and reducing the number of bacteria using its bactericidal capabilities. Moreover, it also may be an indicative of a strong antimicrobial effect as a result of the strong oxidative properties of PTS used in the present study. The presence of organic matter, including proteins in the abdominal cavity, may interfere with the effect of PTS. Previously, organic matters were reported to reduce the antimicrobial activity of the plasma since they react and consume reactive species produced by plasma with antimicrobial properties. Thus, despite the organic matter load in the abdominal cavity, achieving a strong antimicrobial effect suggests that PTS contains and delivers a large amount of antimicrobial reactive species, which maintains the effectiveness of PTS used in the present study in the presence of organic substances^31,51^.

Sepsis is characterized by systemic inflammation that occurs as a response to infection. In the case of sepsis, activated leukocytes release reactive oxygen radicals and proteases that lead to a reduction in antioxidant levels, disturbing the oxidant/antioxidant balance and creating oxidative stress^52,53^. The measurement of various oxidant or antioxidant molecules can be performed separately, but this approach is labor-intensive, time-consuming, costly, and impractical. The severity of oxidative stress is dependent on the status of both oxidants and antioxidants, with a positive correlation with oxidants and a negative correlation with antioxidants. Based on these correlations, the result of a practical calculation obtained by dividing TOS value with TAS value was named as oxidative stress index (OSI), and this formula has frequently been used in either experimental or clinical conditions when TOS and TAS values are present. Since the quantification of TOS and TAS as well as the calculation of OSI allow us to be informed regarding the presence and severity of oxidative stress, together with the status of the counteracting antioxidative system, we used these parameters to assesses the effects of PTS on the abdominal originated sepsis.

Oxidative stress in sepsis has been widely investigated by many researchers worldwide. The total antioxidant capacity is a reliable marker to indicate sepsis-induced oxidative stress, and it has been shown to be lower in patients with sepsis^54^. In a former clinical study by these researchers, no change was observed in total antioxidant capacity in a patient with severe sepsis that resulted in death^55^. Akbulut et al. demonstrated that TAS values in the CLP group were lower than those in the Sham group in their research, to determine the variations in acute phase reactants in rats within the sepsis model^56^. In Tavasoli et al. study, although antioxidant enzymes were high in the treatment group after sepsis induction, lower TAS values were observed in this group^57^. Pascual et al. investigated the total plasma antioxidant capacity and found that it is decreased in septic patients compared to control patients but increased in patients with septic shock^58^. They stated that the increase in some oxidants contributed to an increasing total antioxidant capacity in patients with septic shock. Our study results revealed that the TAS value of Group CPL + Plasma was lower than that of Group Sham. The findings of the present study are in agreement with those of other authors. The decreased TAS in CPL and CPL + Plasma groups is probably due to the consumption of the antioxidants in infection/sepsis. The higher values in the CPL + Saline group compared to the CPL group may be the effect of the impairment of the integrity of the peritoneal mesothelium and the augmentation of proinflammatory mediator response by aggressive irrigation with saline as also shown in Yao et al.’s study or a compensating mechanism for depleted antioxidative components as mentioned in Lorente et al. study^59,60^.

To analyze the effects of plasma on healthy rats, we first investigated its influence on the production of ROS, which may contribute to the starting or intensification of any inflammation. Our results state that PTS has no short-term adverse effect on rats since there was no difference with respect to the oxidation indexes between the Sham and Plasma groups. Moreover, histopathological results revealed no difference in inflammation and apoptosis scores in lungs and spleen tissues between these groups. As in Zhai et al. study, we also demonstrated that a CLP operation resulted in increased oxidative stress and reduced anti-oxidative ability by depletion of TAS and a trend of elevation of TOS in the CPL group^53^. While achieving a strong antimicrobial effect in the abdominal cavity with the application of PTS, lack of toxicity could be linked to the selectivity of the plasma and plasma-treated liquids on prokaryotic and eukaryotic cells. Several studies reported similar selectivity of plasma and plasma-treated liquids, where they can effectively inactivate prokaryotic cells with no detectable or negligible toxicity on eukaryotic cells^32,61^. The selectivity of the plasma and plasma-treated liquids is attributed to the size difference between prokaryotic and eukaryotic cells, compartmentalization, and well-developed DNA proofreading and repair mechanisms in eukaryotic cells compared to prokaryotic ones^61^.

Regarding TOS values, Group CPL + Plasma had a significantly lower value compared to all the other groups. The comparison of the calculated OSI revealed that Group CPL + Plasma had significantly lower OSI values than Group CPL and Group CPL + Saline. In Demir et al.’s study, they have found that serum TOS and OSI levels were increased in the sera of septic rats compared to healthy rats, and TOS levels were decreased with the treatment^52^. Cowley et al. stated that total redox capacity could measure the efficacy of treatment in severe sepsis since its low values increase over time when the patient heals from sepsis^62^. In a clinical study by Annagur et al. it was found that TOS and OSI levels are elevated in septic neonates but decrease with treatment to normal levels of the control patients^63^. Thus, one of the treatment goals of sepsis is to reduce the TOS level. As represented in the present study, irrigation of the peritoneal cavity with PTS leads to reduced TOS levels. Reduction of the TOS by PTS irrigation despite its oxidative nature could be linked to the rapid activation and up-regulation of the oxidative stress defense system, which was previously shown both on eukaryotic and prokaryotic cells^19,64^.

Apoptosis is an essential mechanism of the body to limit the cells by removing them in a controlled manner. It facilitates the elimination of the stressed cells triggered by intrinsic pathways or the initiation of extrinsic pathways mainly activated by macrophages. The TUNEL assay reveals the extent of DNA fragmentation as a measure of apoptosis in vitro. In Liu et al. study, a higher blood bacterial number was observed to be proportional to the bacterial load in the abdominal cavity^65^. These bacteria absorbed in the peritoneum migrate to the lungs as well as to all other organs and directly interact with phagocytes, which results in cell death and tissue injury^66^. Many studies showed that the sepsis model with CLP increases inflammation in the lung with massive neutrophil infiltration, interstitial edema, congestion, and alveolar septum thickening in rats^53,67^. Hotchkiss et al. reported an increase of splenic lymphocyte apoptosis in septic mice, which is also associated with the severity of the disease^68,69^. In our study, we obtained the same results. The histology and TUNEL scores were the same in the Sham and Plasma groups and higher in CPL groups compared to these groups.

Animal experiments of intraabdominal lavage after CPL have shown either beneficial or adverse effects^70,71^. Some studies with saline irrigation demonstrated results as good as those with antiseptic solutions, but Dunn et al. declared that saline increased bacterial proliferation and mortality rate in rats with intraabdominal infection^72,73^. There is evidence that lavage reduces the number of bacteria that adhere to mesothelial cells, but this effect is transient and is followed by an increase in bacterial populations in a timely manner^44,74^. The reason for the adverse effect of irrigation is assumed to be that lavage can remove inflammatory mediators such as complement proteins, proteases, opsonins, and immunoglobulins and cause more injury to the mesothelial cell membranes^75,76^. Nunes et al. reported lesser inflammation in lungs after CPL-induced sepsis in rats but not much difference in splenic specimens^77^. Our results revealed that the CPL + S group showed higher inflammation and apoptosis scores than the Sham group, but the lower apoptosis scores in both organs compared to the CPL group led us to consider the protective effect of saline irrigation as well.

In light of these controversial results with saline irrigation, some researchers investigated the effect of enhanced saline with oxidizing agents or ions or saline with acidic pH levels to reduce the bacterial load in the peritoneum. Kubota et al. used electrolyzed strong acid water containing various chlorine species that are also present in PTS, to perform peritoneal irrigation in a CPL model; they found that the bacterial count was significantly lower in this group compared to the Saline group. The survival rate was also higher in this group^28^. In Ozmen et al., ozonated saline was found as the most effective irrigating solution for reducing abscess formation in survivors after intraabdominal contamination compared to normal saline and saline-cephalothin irrigation^30^. The effects of hydrogen-rich saline have been shown to have potential protective effects by reducing oxidative stress and apoptosis in a rat model of CPL-induced sepsis^78^. Other researchers found less alveolar thickening and neutrophil numbers in the lung, lower inflammatory cytokine levels, and less apoptosis after irrigation with hydrogen-rich saline^29,53^. In our study with PTS, we found similar results. PTS irrigation declares the lowest histology and TUNEL scores compared to all the other CPL groups with no difference of Sham groups, which might demarcate it as an effective technique to minimize sepsis-induced organ injury. The lack of apoptosis in the PCL-Plasma group despite the oxidative feature of PTs could be attributed to the selectivity of cold plasma and plasma-treated liquids on apoptosis between cancer and healthy cells^79,80^.

To conclude the present study, the plasma-treated solution was tested for its application in a rat acute peritonitis model, to prevent sepsis and provide intraabdominal decontamination. Prior to the in vivo experiments, the chemical and in vitro microbiological evolutions were performed. The 45-min PTS can effectively lead to 7-log inactivation of gram-negative and positive bacteria. One of the standard clinical practices in case of acute peritonitis is the irrigation of the peritoneal cavity with a saline solution. In the present study, the irrigation of the peritoneal cavity with PTS led to the decontamination of the cavity and prevented sepsis, with no sign of toxicity. Similarly, in a previous study in which the direct plasma treatment was applied on the peritoneum for the prevention of peritoneal adhesions, cold plasma showed no short-term local and systemic toxicity^22^. Lack of toxicity despite the strong acidity of 45-min plasma treated saline solution seems to be interesting. In a previous recent study, where a plasma treated liquid with acidic pH was injected in rats at large quantities and no toxicity that could be attributed to acidic pH was not also reported^81^. Such findings could be attributed to presence of excessive organic load in vivo which could be acting as a buffer that brings the pH to a level where it wouldn’t cause toxicity at the site of plasma treated fluid (in our case 45-min PTS) application. The promising effects of PTS in acute peritonitis could be linked to ROS in the PTS that was created during the plasma treatment. Irrigation of the peritoneal cavity with the PTS instead of the saline solution could be considered as an effective and potential method to be opt for in case of acute peritonitis in the future.

## Methods

### Plasma treatment of saline solution

**Figure 10:**
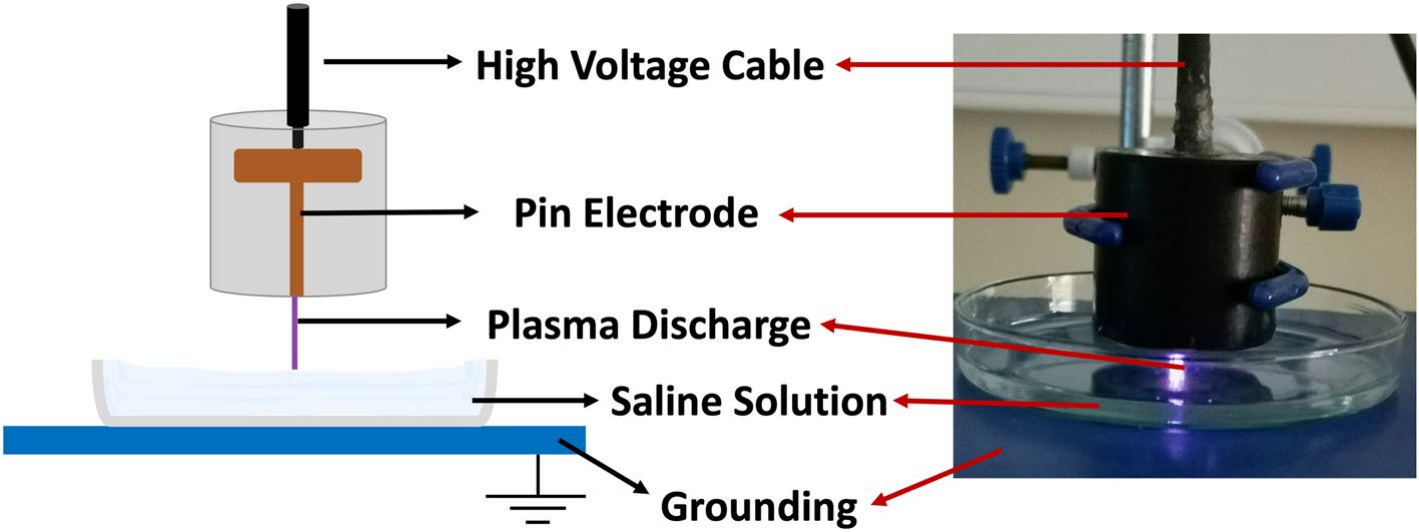
Image and schematics of the plasma setup for the treatment of saline solution.

The sterile saline solution (0.9% NaCl) was treated using a pin-to-liquid discharge set up in ambient air (see Fig. 10). The setup consisted of a copper pin, where the transient spark discharge was obtained, which was connected to a high-voltage cable. Electrical characterization of the plasma treatment setup was measured using a high-voltage probe (Tecpel, Munich, Germany) and a current probe (Tektronix, Beaverton, OR, USA), which were connected to a digital oscilloscope (GW-Instek, GDS-2202A, Taiwan) (Fig. 1A). The microsecond alternating current (AC) power supply was operated at 24 kV peak-to-peak voltage, 3.5 mA discharge current, and 25 kHz frequency, which yielded about 9 W in terms of power output. 15 mL of the saline solution was transferred to a 90 mm-diameter petri dish made of glass and treated for 15–30 and 45 min at a fixed discharge gap of 1.5 mm. After the plasma treatment, the PTS solution was collected and used in further experiments.

### Optical emission spectroscopy (OES)

The optical emission spectra of the pin-to-liquid plasma discharge were recorded with the help of a broad spectral range spectrometer (Aseq Instruments, LR1, Vancouver, BC, Canada). The spectral range and the resolution of the spectrometer were 190–1100 and 2 nm, respectively. The fiberoptic probe of the spectrometer was fixed 5 mm away from the discharge. Emission spectra were collected every 30 s for 10 min, and their average was recorded. The data were analyzed using MATLAB and plotted on Microsoft Excel.

### pH measurement of PTS solution

After each plasma treatment session, 15 mL of the PTS was collected in a centrifuge tube, and its pH was measured with a digital pH meter for each plasma treatment time point (IsoLab, Germany). pH measurements were repeated ten times for each plasma treatment time point.

### Bacterial cultures and in vitro antibacterial tests

*Escherichia coli* American Type Culture Collection (ATCC) 25922 and *S. aureus* ATCC 25923 were used as gram-negative and gram-positive model organisms, respectively, for in vitro antibacterial experiments in the present study. Frozen stocks of each strain were thawed, and 1 mL of the culture was mixed with 9 mL of a trypticase soy broth (TSB) and incubated in a shaker incubator at 37 °C and 120 revolutions per minute (rpm) overnight. Overnight grown cultures were spread on trypticase soy agar (TSA) plates and incubated overnight. Single colonies were collected and homogenized in 10 mL TSB and incubated overnight whenever needed in antibacterial tests. In the present study, a bacterial growth kinetics test and a colony counting assay were utilized to determine the antibacterial efficacy of PTS. In the growth kinetics test, saline solution was treated with plasma for 15, 30, and 45 min. 40 µL of 10^7^ CFU/mL of each strain was mixed with 160 µL of PTS in a 96-well plate. The 96-well plate was placed in a microplate reader (BioTek, Synergy HTX Plate Reader, Winooski, VT, USA), and the absorbance of bacteria with PTS was recorded at 600 nm every 20 min for 18 h to obtain growth curves. Untreated PTS was used as a control. Measurements were repeated three times in triplicate, and the mean of absorbance from each experiment was plotted for PTS treated at different time points.

In the colony counting assay, similarly, 40 µL of 10^7^ CFU/mL of each strain was mixed with 160 µL of PTS (treated at different time points) in a microcentrifuge tube and held for 2 h. Afterward, bacteria were appropriately diluted using 1X sterile PBS, and the diluted cultures were placed on TSA plates. The TSA plates were incubated for 24 h. After incubation, the surviving colonies were counted, and the results were expressed as log-surviving colonies. All plates were incubated for an additional period of 48 h to rule out possible dormancy. Untreated saline solution was used as a control. Experiments were carried out three times in triplicate.

### KI-starch assay

The KI-starch assay was performed to determine the total oxidative strength as an indicator of ROS in PTS. Deionized water (DIW) was heated close to the boiling point, and 60 mg starch was added in 10 mL DIW and stirred to completely dissolve the starch. Then, the starch solution was left to cool, following which 120 mg KI was added to the starch solution and stirred well to dissolve the KI. The saline solution was treated with plasma for 15, 30, and 45 min, as explained. 500 µL of the KI-starch solution was mixed with 500 µL PTS (treated at different time points), and the absorbance was measured at 680 nm using a spectrophotometer (PG Instruments Limited, UK). Untreated saline was used as a negative control, and measurements were repeated three times in triplicate.

### Detection of singlet oxygen

1,3-diphenylisobenzofuran (DPBF) reagent (Sigma Aldrich, St Louis, MO, USA) was used to detect singlet oxygen (^1^O_2_) in PTS. DPBF is a yellow-colored reagent that turns to colorless o-dibenzoyl benzene when oxidized by singlet oxygen^82^. Thus, DBPF could be used to detect singlet oxygen in PTS by monitoring the absorbance change, where lower absorbance would indicate higher concentrations of singlet oxygen. 4 µL of 8 mM DPBF was added to 200 µL of PTS (treated at different time points), homogenized by gentle shaking, and absorbance was recorded between 380 and 500 nm. Furthermore, a 45-min PTS solution was mixed with DPBF, as explained, and absorbance measurement was made after 5, 10, 15, 20, 25, and 30 min of delay. Untreated saline solution was used as a control, and measurements were performed three times in triplicate.

### Detection of nitrite

Griess reagent (Biotium, Fremont, CA, USA) was used to detect nitrite (NO_2_^−^) in PTS. Sulfanilic acid in Griess reagent is converted to a diazonium salt when it reacts with nitrite at acidic pH, which couples with *N*-(1-naphthyl) ethylenediamine to form azo dye that can be detected spectrophotometrically^83^. The saline solution was treated for 15, 30, and 45 min as described. 50 µL of PTS for each time point was mixed with 6.7 µL Griess reagent, and the total volume was brought to 100 µL by adding DIW. Then, the mixture was held at room temperature for 30 min, and absorbance measurement was performed at 548 nm.

### Animals

The study was conducted at the Experimental Animal Laboratory of Ege University following approval from the Animal Experiments Ethics Committee of the same institution (#2019-034). All procedures in this study complied with the National Institutes of Health Guide for the Care and Use of Laboratory Animals recommendations (publication no. 86-23, revised 1985, Bethesda, MD) and ARRIVE guidelines. 60 adult female Wistar albino rats weighing 200–250 g were used in the study. All animals were housed separately under 12 h light/dark circles and a constant temperature (20–24 °C) and humidity (60–70%). Standard rat chow and water were given to them ad libitum.

### Experimental model

Following 12-h fasting, the rats were randomly divided into six groups. The first group was the sham-operated group (Group Sham, n = 10). The animals underwent laparotomy and cecal mobilization at time zero and after 2 h and were sacrificed at the end of the 6th h. The second group was the Plasma group (n = 10). The sham operative procedure was performed, and after 2 h, the abdominal cavity was irrigated with 10 mL of body-warm PTS solution three times for 5 min for each irrigation. The abdomen was dried and closed. In the CLP group (n = 10), the animals were submitted for laparotomy, and cecal ligation and puncture (CLP) was applied. After 2 h, the ischemic cecum was resected. In the CLP + Dry cleaning group (n = 10), the same procedure was applied, but all areas in the abdominal cavity were also cleaned with a sterile gauze two hours after CLP was performed. In the CLP + Saline group (n = 10), laparotomy and CLP were performed. After 2 h, the ischemic cecum was resected, and the abdominal cavity was irrigated with 10 mL of body-warm 0.9% saline solution three times for 5 min for each irrigation. The abdomen was dried and closed. In the CLP + Plasma group (n = 10), laparotomy and CLP were performed. After 2 h, the ischemic cecum was resected. The abdominal cavity was irrigated with 10 mL of body-warm PTS solution three times for 5 min for each irrigation. The abdomen was dried and closed. The PTS that was used for the irrigation of the peritoneal cavities of rats was prepared the day before the surgery and stored at 4 °C for 18–24 h. Right before the irrigation of peritoneal cavities, the temperature of the PTS was brought to 37 °C by keeping it in an incubator for 10 min. Six hours after the irrigation of the peritoneal cavity, animals were sacrificed by heart puncture which is also used to collect blood samples, and blood and tissue specimens were harvested for analysis.

### Cecal ligation and puncture model

The cecal ligation and puncture (CLP) model described by Wichterman et al. was used in this study^84^. The rats were anesthetized with an intramuscular injection of 70 mg/kg ketamine HCl (Ketalar®, Parke-Davis, Michigan, USA) and 10 mg/kg xylazine HCl (Rompun®, Bayer, Germany). Surgery was performed under aseptic conditions. The anterior abdominal wall was shaved, and the skin was disinfected with iodine solution. The abdominal cavity was entered through a 2-cm midline incision. The caecum was ligated with a 3–0 silk suture below the ileocecal valve without causing intestinal obstruction. The ligated portion was punctured twice with a 21-gauge needle, and this portion was squeezed gently to expel a small amount of fecal material through the puncture holes. The cecum was placed back into the abdominal cavity. The layers of the abdominal wall were closed appropriately with 4–0 prolene.

### Biochemical evaluation

Serum total antioxidant status (TAS) was measured using the 2,2′-azino-di-3-ethylbenzthiazoline sulfonate^+^ colorimetric method. Total oxidant status (TOS) was evaluated using a novel automated and colorimetric measurement method on AU5800 autoanalyzer (Beckman Coulter Inc., CA, USA)^85^. The TAS results were expressed as μmol Trolox equivalents/L, and the TOS results were expressed in terms of micromolar hydrogen peroxide equivalent per liter (μmol H_2_O_2_ equiv./L). The ratio of TOS level to TAS level was calculated as the oxidative stress index (OSI).

### Microbiological evaluation

5 mL of venous blood was collected with heart puncture under sterile conditions. Venous blood samples were restored in an anaerobic blood culture vial (BD Peds Plus/F). Pediatric blood culture vials were used in case the samples taken for aerobic blood culture were less than 3 ml. After the sample to be tested was inserted into the vial, the vials were placed in an automated blood culture device (BD BACTEC) for incubation and periodic reading. Blood samples were incubated for five days in the BACTEC FX (BD, USA) automated system. Each vial had a sensor that would detect the increase in CO_2_ caused by microorganism growth. The sensor was monitored by the instrument every 10 min for an increase in its fluorescence; this increase is proportional to the amount of CO_2_ available. A positive reading is predictive of finding viable microorganisms in the vial. Aerobic and anaerobic bottles that gave positive signals during incubation were plated on Columbia agar for anaerobic culture and 5% sheep blood agar (Salubris) and eosin methylene blue (EMB) agar and chocolate agar for the growth of aerobic bacteria. Cultivated anaerobic media were placed in an anaerobic jar, and an oxygen-free environment was provided with a dry system gas package (AnaeroGen-Oxoid, Basingstoke, UK). Anaerobic indicator (Oxoid, Basingstoke, UK) was used as an indicator to control the anaerobic environment. To produce anaerobic bacteria, the media were incubated in an anaerobic environment at 35–37 °C for 48 h.

The incubation time was 24–48 h for aerobic bacteria. At the end of the evaluation, results that appeared contaminated were excluded from the study. Passages were made to EMB agar, 5% sheep blood agar, and chocolate agar media and incubated for 24–48 h at 37 °C. The identification of aerobic microorganisms was done according to colony morphology and Gram staining characteristics using conventional methods (Coagulase in the tube, PYR test, growth in 6.5% NaCl, oxidase test, and biochemical tests (triple sugar iron agar, urea agar, motility medium, and reactions in indole medium) and using an automated system (Phoenix, BD, USA).

At the end of the incubation, anaerobe agar and chocolate agar were plated from each colony growing in anaerobic media, and it was checked whether the bacteria were aerobic or aerotolerant. Bacteria that grow in an anaerobic environment but do not grow on chocolate agar in a 5–10% CO_2_ environment were evaluated as anaerobic bacteria. After the differentiation of anaerobic bacteria from facultative anaerobic bacteria, pure cultures were obtained by making their passages in anaerobic conditions. Anaerobe identification was not necessary due to the absence of anaerobic growth.

### Histopathological evaluation

Specimens of the lung and spleen were harvested and immediately fixed in 10% formaldehyde for at least 24 h. The fixed tissues were dehydrated in graded ethanol and embedded in paraffin. 5 µm-thick sections were cut on a rotary microtome (Leica RM2245) and stained with hematoxylin and eosin (H&E). A scoring system was used to assess the degree of lung injury based on the following histological features: edema; hyperemia and congestion; neutrophil margination and tissue infiltration; intraalveolar hemorrhage and debris; and cellular hyperplasia. These characteristics were subjectively scored on a scale between 0 and 3 (0 = normal; 1 = slight effect; 2 = moderate presence of that feature; and 3 = severe effect)^86^. Spleen samples were analyzed for the severity of polymorphonuclear neutrophil (PMN) infiltration, and indexes were scored as 0 (normal), 1 (mild), 2 (moderate), and 3 (severe)^87^.

For the distribution of apoptotic cells, the terminal deoxynucleotidyl transferase dUTP nick end labeling (TUNEL) assay was used according to kit procedure (S7101, ApopTag, Millipore). All samples were fixed in 10% formalin for 24 h and then processed for embedding in paraffin using a routine protocol. The paraffin sections were deparaffinized in xylene and incubated with 20 µg/ml of proteinase K for 15 min before being rinsed with PBS. Endogenous peroxidase was inactivated with 3% hydrogen peroxide for 5 min. Then, the samples were incubated with an equilibration buffer for 5 min, and the TdT enzyme was added following incubation in a humidified atmosphere at 37 °C for 60 min. Samples were washed with a stop/wash buffer for 10 min, and the anti-digoxigenin conjugate was added and incubated for 30 min at room temperature. After each step, the slides were washed with PBS. Staining was done with peroxidase substrate, and counterstaining was performed in Mayer’s hematoxylin. After mounting with entellan, the sections were examined independently by two histologists, and the cells were counted on randomly chosen fields per case to check whether they are TUNEL-positive or not. The percentage of TUNEL-positive cells with positive brown staining was determined. Two observers blinded to treatment assignment assessed the staining scores independently. TUNEL-positive cells were scored based on the percentage of positively stained cells, as follows: Grade 1 = 10% of the cells are positive, Grade 2 = 10% but 50% of the cells are positive, and Grade 3 = 50% of the cells are positive^88^.

### Statistical analysis

The data analysis was carried out using IBM SPSS Statistics for Windows (version 21.0; IBM Corp., Armonk, NY, USA). The results were presented as mean ± standard deviation. The normal distribution of the numerical variables was determined using the Shapiro–Wilk normality test. If the data complied with a normal distribution, the statistical differences between the groups were evaluated using the one-way analysis of variance and post hoc tests. If the data did not comply with a normal distribution, Mann–Whitney U tests were used. A *p*-value of < 0.05 was considered in determining statistical significance.
